# Chlorogenic acid inhibits virulence and resistance gene transfer in outer membrane vesicles of carbapenem-resistant *Klebsiella pneumoniae*

**DOI:** 10.3389/fphar.2025.1562096

**Published:** 2025-03-31

**Authors:** Wen-Ba Wang, Jia-Yang Wan, Dao-Jun Yu, Hai-Xia Du, Hui-Fen Zhou, Hai-Tong Wan, Jie-Hong Yang

**Affiliations:** ^1^ College of Basic Medicine, Zhejiang Chinese Medical University, Hangzhou, China; ^2^ The First Affiliated Hospital, Zhejiang University School of Medicine, Hangzhou, China; ^3^ Department of Medical Laboratory, Affiliated Hangzhou First People’s Hospital, Zhejiang University School of Medicine, Hangzhou, China

**Keywords:** outer membrane vesicle, carbapenem-resistant *Klebsiella pneumoniae*, resistance gene transfer, cell pyroptosis, chlorogenic acid

## Abstract

**Introduction:**

Carbapenem-resistant *Klebsiella pneumoniae* (CRKp) infection poses a significant global public health challenge, with the misuse of antibiotics further contributing to the development of resistance and triggering harmful inflammatory responses. Outer membrane vesicles (OMVs) released by CRKp under sub-lethal concentration of MEM pressure (KOMV-MEM) exhibit enhanced virulence and greater efficiency in transferring resistance genes.

**Methods:**

We investigated the inhibitory effects of chlorogenic acid (CA) on KOMV-MEM characteristics and its protective role in KOMV-MEM infected mice. Based on LC-MS proteomic analysis of vesicles, we screened for potential targets of KOMV-MEM in promoting macrophage (MØ) pyroptosis pathways and inducing resistance gene transfer. Subsequently, computational predictions and experimental validation were performed to determine how CA regulates these mechanisms.

**Results:**

This study confirmed that, under MEM pressure, the exacerbated infection levels in CRKp-inoculated mice are attributable to the high virulence of KOMV-MEM. Computational and experimental results demonstrated that CA inhibits pyroptosis by reducing MØ capture of KOMV-MEM through blocking the interaction between GroEL and LOX-1. Furthermore, CA prevents the spread of resistance genes by disrupting the conjugation and transfer processes between KOMV-MEM and recipient bacteria. Finally, in vitro and in vivo assays showed that CA inhibits KOMV-MEM resistance enzymes, thereby preventing the hydrolysis of MEM in the environment and depriving susceptible bacteria of protection.

**Discussion:**

These findings provide the first confirmation that CA can inhibit both the virulence and the transmission of drug resistance in KOMV-MEM. This underscores the potential of CA treatment as a promising antimicrobial strategy against CRKp infection.

## 1 Introduction

Antibiotic resistance involving pathogens has become a global public health concern requiring urgent attention (Laxminarayan et al., 2013). Carbapenems, due to their extensive antimicrobial spectrum and capacity to withstand β-lactamases, are frequently used to manage recalcitrant infections, including urinary tract infections, pneumonia, meningitis, and bacteraemia. Thus, they are considered the “last resort” for treating infections caused by resistant bacterial strains (Galbadage et al., 2019). However, misuse of antibiotics has gradually led to a breach of this last line of defence (Wang et al., 2024). Increasing incidences of multidrug-resistant (MDR) bacterial infections have been reported, including those caused by carbapenem-resistant *Klebsiella pneumoniae* (CRKp) (Wyres et al., 2020). Pathogens have evolved unique mechanisms, such as efflux pumps, biofilm formation, and enzyme-mediated reactions, to survive under adverse conditions, including antibiotic pressure and host immune responses. These mechanisms play a significant role in the development of drug resistance (Upadhayay et al., 2023). It has long been believed that the pathogenicity genes of highly virulent *K. pneumoniae* (hvKp) and antibiotic resistance genes of CRKp are distributed among different subpopulations of *K. pneumoniae* (Wyres et al., 2019). However, evidence suggests that this balance can be disrupted (Lam et al., 2019). hvKp and CRKp can combine to form the highly virulent CRKp (hv-CRKp) through plasmid transfer (Yang et al., 2021; Tian et al., 2022).

Although antibiotics are the cornerstone of antimicrobial treatment, the absence of swift diagnostic tools for identifying bacterial resistance phenotypes in clinical settings, particularly in less developed areas owing to technological and financial constraints, culminates in the misuse and overuse of these critical drugs (Bassetti et al., 2018). Beyond the intrinsic adverse effects of antibiotics, their misuse in infection management is not a “cost-free scenario.” In a clinical investigation focused on necrotizing pancreatitis suggest that imprudent administration of antibiotics is an independent determinant of enterococcal bacteraemia-related mortality (Timmerhuis et al., 2023). Considering the paucity of comprehensive clinical data, a prudent appraisal of these outcomes is imperative. Gram-negative bacteria release extracellular lipid vesicles, termed outer membrane vesicles (OMVs), via a series of processes, including outer membrane blebbing, inner membrane protrusion mediated by autolysins, and “explosive cell lysis” (Toyofuku et al., 2019). OMVs, characterised by their spherical morphology and a size range of 20–300 nm, are delineated by a single or double lipid bilayer, encapsulating an array of potential cytoplasmic constituents, such as plasmid DNA, RNA, and proteins (Turnbull et al., 2016). OMVs also carry membrane-expressed proteins, which are crucial virulence factors, contributing significantly to the bacteria’s pathogenicity by facilitating interactions with host cells and modulating immune responses (Li et al., 2022; Li et al., 2025). Functionally, these vesicles are implicated in the long-distance horizontal transfer of lipopolysaccharides (LPS), virulence factors, and antibiotic resistance genes to recipient bacteria or cells, thereby safeguarding the enclosed materials from degradation by the external environment (Liu et al., 2018). Critically, in the presence of sublethal antibiotic exposure, MDR bacteria manifest significant outer membrane perturbations, including envelope crosslinking, peptidoglycan fragment accumulation, erroneous protein folding, autolysin-induced cellular lysis, and membrane reconstitution (Toyofuku et al., 2019; Ye et al., 2021; Fulsundar et al., 2014). Such disturbances precipitate a pronounced escalation of both the release of OMVs and their associated virulence. These findings offer new insights into the hazards associated with antibiotic misuse. Current strategies predominantly focus on direct bacterial killing or resistance gene suppression, yet fail to address the ‘stealth threat’ posed by antibiotic-stimulated OMVs.

**FIGURE 1:**
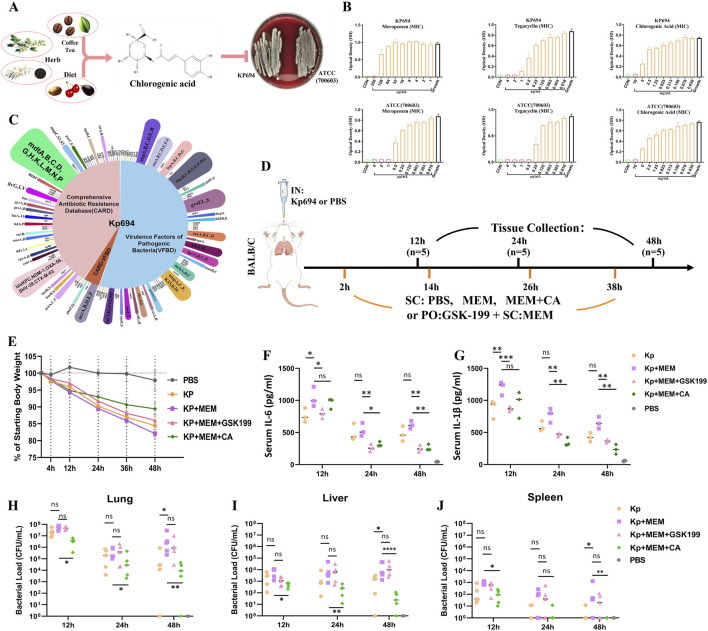
Biological characteristics of Kp694 strain and the *in vitro* and *in vivo* antibacterial effects of CA. **(A)** Chart showing the source of CA and its antimicrobial action. **(B)** MIC of Meropenem, Tigecycline, and CA for Kp694 (n = 3); MIC of Meropenem, Tigecycline, and CA for ATCC 700603 (n = 3). **(C)** Annotation Results of Kp694 Coding Genes Using VFBD and CARD Annotation Results of Kp694 Coding Genes by VFBD and CARD. **(D)** Animal experimental protocol. **(E)** Weight after infection (n = 5). **(F, G)** IL-6 and IL-1β concentration in the serum fluid, measured using ELISA (n = 3). **(H–J)** Bacterial load in Lungs, Liver and Spleen were estimated by serially diluting samples and plating onto MEM-containing agar plate (n = 5). All data are expressed as mean ± S.D. *p < 0.05, **p < 0.01, ***p < 0.001.

With traditional antibiotics losing efficacy against CRKP, alternative strategies are being explored. Chlorogenic acid (CA), a phenolic compound characterised by various isomeric forms, of which 5-O-caffeoyl-D-quinic acid is the most prominent, is abundant across a spectrum of dietary sources (including coffee beans, green tea, cherries, and eggplants) and medicinal herbs (such as honeysuckle and cassia seeds) (Tajik et al., 2017). The chemical structure of CA is shown in Figure 1A. Its extensive examination in the scientific literature can be attributed to its early identification and presentation in the crystalline form (Lu et al., 2020). CA is a natural agent with antimicrobial and antiviral properties, demonstrating broad-spectrum activity against various pathogens, including viruses, bacteria, fungi, and amoebic protozoans (Lou et al., 2011). Notably, CA can compromise bacterial membrane integrity without inducing haemolysis, thereby enhancing membrane permeability and leading to the dissipation of membrane potential and subsequent cellular leakage (Gou et al., 2023; Ruifeng et al., 2014). However, to date, there is no report on the effects of CA on virulence and horizontal gene transfer of bacterial OMVs, especially against the backdrop of the “silent pandemic” of MDR bacteria, where antibiotics have shown limited efficacy (Sivanantham et al., 2023). Therefor this study first explored the mechanism through which CA influences the virulence and horizontal gene transfer of OMVs released by CRKp under the stimulation of meropenem (MEM).

## 2 Materials and methods

### 2.1 Bacterial strains and growth conditions

The clinical isolate *K. pneumoniae* (Kp694) and the ATCC700603 quality control strain both come from the First People’s Hospital of Hangzhou, Zhejiang Province, China. The clinical isolates were subjected to whole-genome shotgun sequencing to create libraries of varying insert sizes. Next-generation sequencing, specifically paired-end sequencing, was used on the Illumina NovaSeq platform for these libraries. The sequencing data for Kp694 have been submitted to the National Centre for Biotechnology Information database (GenBank accession number: PP348266). All strains were cultured at 37°C in lysogeny broth (LB) medium (200 r/min), LB agar plates, or Columbia blood agar plates.

### 2.2 THP-1 MØ culture

We employed THP-1 cells (Servicebio, China) at passage eight for macrophage induction (Liu et al., 2019). THP-1 cells were cultured in RPMI-1640 medium (Servicebios, China) containing 10% fetal bovine serum (FBS; Servicebios, China) and 1% penicillin–streptomycin, in a humidified incubator at 37°C with 5% CO_2_. To induce MØ, THP-1 cells are treated with 30 ng/mL Phorbol 12-myristate 13-acetate (PMA) (Sigma-Aldrich, USA) for 72 h, followed by stabilization in fresh medium for 24 h. All cells are cultured in a humidified incubator at 37°C with 5% CO_2_.

### 2.3 CCK-8 assay

A total of 8 × 10^3^ MØ cells per well were seeded into 96-well plates and allowed to adhere for 24 h. The cells reached approximately 80% confluence, they were treated with various concentrations of CA (Shanghai yuanye, China) at 37 °C for 24 h. Following this incubation, CCK-8 solution (Servicebio, China) was added, and the plates were further incubated at 37°C for 2 h. The optical density (OD) at 450 nm was then measured to assess cell viability.

### 2.4 Preparation and protein quantification of Klebsiella-derived OMVs (KOMVs)

Individual Kp694 colonies were placed in fresh LB (with or without MEM and CA) and incubated at 37°C, 200 r/min for 18–20 h to achieve an optical density (OD600) of approximately 0.9. The culture fluid (250 mL) was collected and centrifuged at 3,000 g for 20 min and at 12,000 g for 45 min, and thereafter, filtered through a 0.22-μm aqueous membrane filter. The precipitate was concentrated twice by ultracentrifugation using an Optima XPN-100 (Beckman, USA) at 4°C and 120,000 g for 2 h. The concentrated OMVs were adjusted to a volume of 2 mL with 50% Optiprep (Sigma-Aldrich, USA) HEPES, placed at the bottom of a gradient tube, layered with 2 mL each of 40%, 30%, 25%, and 15% Optiprep HEPES, and centrifugated at 4°C and 100,000 g for 8 h. Thereafter, the gradient fractions were removed from the top in 2 mL aliquots and identified sequentially, as previously described (Unni et al., 2021). The fractions containing OMVs were combined and diluted with HEPES, then centrifugated at 4°C and 120,000 g for 2.5 h. The washing was repeated twice with HEPES. OMV particles were resuspended in HEPES, and their sterility was verified on agar plates. The various groups of OMVs (KOMV, KOMV-MEM, and KOMV-MEM-CA) were treated with 2 U DNase I (Servicebio, China) and stored at −80°C for future use. The total protein concentration of OMVs was determined using the Bradford protein assay kit (Thermo Fisher Scientific, United States).

### 2.5 Animal studies

The animal experiment protocol was reviewed and approved by the Ethics Committee for Laboratory Animal Welfare of Zhejiang University, complying with animal welfare and ethical principles, and adhering to the relevant regulations on laboratory animal welfare and ethics in China (Approval CODE: ZJU20240701). All procedures were conducted in biosafety level-2 facilities. Female BALB/c mice aged 6–8 weeks, free of specific pathogens, were obtained from the Laboratory Animal Center of Zhejiang University. Mice were housed in individually ventilated cages at a density of five mice per cage and allowed free access to food and water. All cages were kept in the same specific pathogen-free (SPF) room, with room temperature maintained at 20°C–24°C, humidity controlled at 55% ± 10%, and a 12-h light/dark cycle was used. For model establishment, after inhalation anesthesia with isoflurane, the mice were intranasally instilled with the following solutions: 50 µL of *K. pneumoniae* (Kp694 or ATCC700603) at a concentration of 5 × 10^7^ CFU/mL, 50 µL of OMVs at a concentration of 2 × 10^12^ particles/mL, or 50 µL of PBS (Biological Industries, Israel) (control group). For the dosing regimen, the mice were subcutaneously injected (SC) with solutions (maximum single dose volume 100 µL); the single dose was as follows: 50 mg/kg MEM (Auonsi, China), 20, 40, or 80 mg/kg CA (Aladdin, China), or PBS (control group). Mice were orally administered solutions (maximum single dose volume 200 µL); single doses were: 10 mg/kg GSK-199 (AbMole, USA). Weight changes and survival were monitored after mouse inoculation. Finally, the mice were euthanized by intraperitoneal injection of sodium pentobarbital. Blood was collected from the retro-orbital plexus, allowed to clot at room temperature, and centrifuged at 4°C to obtain serum. The lungs, liver, and spleen were collected from euthanized mice and stored in a −80°C freezer for future experiments.

### 2.6 Bacterial load assay

The lungs, liver, and spleen were dissected and homogenized in 1 mL of PBS. The obtained lung, liver, and spleen homogenates were serially diluted with PBS at a ratio of 1:10, and then plated onto MacConkey agar plates (Becton, USA) with or without MEM. The plates were incubated overnight at 37°C, and individual colonies were counted at appropriate dilutions, with the average value taken to determine the colony-forming units (CFU) per milliliter.

### 2.7 Histopathological analysis

Lung tissues were fixed with 4% paraformaldehyde; these were embedded in paraffin sections (2 μm thick), de-paraffinized, and then rehydrated with an ethanol gradient. Lung tissues were treated with hematoxylin and eosin (H&E) for staining and photographed under light microscopy.

### 2.8 OMVs were subjected to proteomic analysis by LC-MS/MS

Add lysis buffer (8M urea + 1% SDS, with protease inhibitors) to the sample at a specific ratio, vortex to mix, and sonicate using an ultrasonic cell disruptor for 2 min. Lyse on ice for 30 min, vortexing every 10 min, then centrifuge at 14000 g for 20 min at 4°C. Collect the supernatant and quantify the protein content using the BCA method. For protein pretreatment, use the iST sample preparation kit (PreOmics, Germany). Mix the protein with 50 μL of lysis buffer, heat at 95°C and 1,000 rpm for 10 min, then cool to room temperature. Add trypsin digestion buffer and incubate at 37°C and 500 rpm for 2 h. Terminate the digestion with stop buffer, and desalt peptides using the iST cartridge, eluting with 2 × 100 µL of elution buffer. Vacuum dry the peptides and store at −80°C. Re-dissolve the lyophilized peptides in 0.1% formic acid and analyze by LC-MS/MS using an UltiMate 3,000 system connected to a timsTOF Pro2 mass spectrometer (Bruker Daltonics). Load 200 ng of sample and use a 90-min gradient for separation at 50°C and a flow rate of 300 nL/min. Operate the mass spectrometer in DDA PASEF mode, scanning from 100 to 1,700 m/z with ion mobility and dynamic exclusion settings adjusted for optimal detection. Analyze the tandem mass spectra using PEAKS Studio version 10.6 with appropriate search parameters for trypsin digestion and modifications. Perform label-free quantification using the software’s alignment and normalization features to compare protein abundance across samples.

### 2.9 Nanoparticle tracking analysis (NTA) and transmission electron microscopy (TEM)

Sample cells were rinsed with deionised water (Particle Metrix, Germany), followed by instrumentation using 100-nm polystyrene beads. Subsequently, the sample cells were rinsed with 1×PBS to ensure system accuracy. OMVs were diluted with 1×PBS and analysed. The collected video data were analyzed using NTA software.

OMVs were observed and imaged using TEM (H-7650, Japan). After staining the OMV samples with 2% uranyl acetate for 3 min, 15 μL of the sample cell was placed on a lacey carbon film, with excess fluid absorbed using filter paper. The morphology of the OMVs was observed using TEM at an operating voltage of 80 kV.

### 2.10 Fluorescent labelling of OMVs and ATCC (700,603) for confocal microscopic observation

Purified OMVs were incubated with the lipophilic membrane dye DIL (Servicebio, China) at 30°C in the dark for 40 min. The labelled OMVs were resuspended in HEPES buffer and centrifuged at 4°C and 120,000 g for 2 h. The step was repeated twice to wash away the free dye and collect the labelled OMVs. ATCC700603 bacterial culture with an OD600 of 0.9 was incubated with an appropriate amount of the lipophilic membrane dye, DIO (Servicebio, China), in the dark at 30°C for 1 h. The bacteria were washed thrice with PBS to remove the free dye and then incubated with DIL-labelled OMVs in the dark at 30°C for 4 h. The bacteria were then washed twice with PBS. Imaging was performed using a 63× oil objective lens with a laser scanning confocal microscope (LSM880, Germany). Images were processed using ImageJ software version 1.54i (National Institute of Health, Bethesda, MD, United States).

### 2.11 Protein interaction and molecular docking

The receptor proteins, including OmpA (PDB ID: 2K0L), TraI-ssDNA (PDB ID: 5N8O), TraN-OmpK36 (PDB ID: 7SZI), GroEL (PDB ID: 8BL2), LOX-1 (PDB ID: 7W5D) were obtained from RCSB Protein Data Bank (PDB, http://www.rcsb.org/) database (Rose et al., 2017). Protein interaction predictions were performed using Alphafold 3 (https://golgi.sandbox.google.com/) (Abramson et al., 2024). The three-dimensional structure of CA was downloaded from the PubChem database (Kim et al., 2021). Subsequently, the MOL2 file was transformed into a PDB format using Open Babel 2.4.1 software. In the current study. First, ligands for docking can be prepared through PyMOL selections, including water deletion and the extraction of original ligands, ions and solvents. Then proteins and ligands were saved through Autodock Tools in PDBQT formats (Bugnon et al., 2024). After docking, the two ligands with the lowest affinity score for the receptors were selected for further analysis. After the docking analysis, the active sites were constrained using Autodock Vina v.1.2.0 software (https://vina.scripps.edu) to enhance the accuracy of SFN binding predictions (Bugnon et al., 2024).

### 2.12 RNA extraction and RT-PCR

Total RNA from bacteria and cells was extracted using the FastPure Cell RNA Isolation Kit V2 (Vazyme, China) and dissolved in 50 μL of nuclease-free water (ddH2O). The RNA concentration was measured using a Nanodrop One microvolume spectrophotometer (USA). Reverse transcription was performed using the Reverse Transcription (with gDNase) kit (biosharp, China). RNA levels were quantified using the 2X Universal SYBR Green Fast qPCR Mix (ABclonal, China) with specific primers (Supplementary Table S1) in a QuantStudio Q3 PCR system (Thermofisher, United States).

### 2.13 Cytokine, LDH and LAL detection

Induced macrophages (MØ) were co-cultured with various concentrations of KOMV, KOMV-MEM, KOMV-MEM-CA (with or without free CA), and the cell supernatants were collected for cytokine or LDH analysis. LDH levels were measured according to the instructions of the LDH Cytotoxicity Assay Kit (Abcam, Britain). The percentage (%) of LDH release was calculated using the formula (Absorbance of OMVs or PBS treated samples/Absorbance of LDH release agent treated samples) × 100%. TNF-a, IL-6 and IL-1β in cell supernatant or serum levels were quantified using ELISA kits (Mlbio, China). After washing with PBS and centrifuging to obtain the cell pellet, cells were lysed using RIPA lysis buffer. A suitable amount of cell lysate was taken and analyzed using the Chromogenic LAL Endotoxin Assay Kit (Beyotime, China), with the experimental steps strictly following the instructions in the kit manual. The reaction solution was measured for OD at 545 nm, and the LPS concentration in the cell cytoplasm was calculated using a standard curve.

### 2.14 SDS-PAGE and coomassie blue staining

OMVs samples of the same particle concentration were mixed with 5× loading buffer and denatured at 95°C for 10 min. Load 10 μL of the sample onto a 12% SDS-PAGE gel for electrophoresis. After electrophoresis, the gel can be stained with Coomassie Brilliant Blue staining solution (Servicebio, China) and destained with deionized water.

### 2.15 Western blot

Cells samples were lysed in RIPA buffer containing protease and phosphatase inhibitors for 30 min. Protein concentrations were measured using the BCA assay kit (Beyotime, China). Proteins were denatured by heating in a 95°C water bath. Electrophoresis was carried out to transfer proteins to PVDF membranes. Membranes were incubated overnight at 4°C with the primary antibodies GAPDH (CST, 5174T, 1:1,000, America), NLRP3 (CST, 15101T, 1:1,000, America), GSDMD (Servicebio, GB115429-100, 1:500, China) then washed thoroughly three times with PBST and incubated with HRP-conjugated secondary antibodies (1:5,000 dilution) for 1 h at room temperature. Bands were detected with ECL (AmershamPharmacia Biotech, Piscataway, NJ) and the intensity of the bands was quantified using the ImageJ gel analysis software.

### 2.16 Immunofluorescence technique

DIL-labelled OMVs were co-cultured with macrophages (MØ) for 6 h. After removing the supernatant and washing with PBS, the sample cells were fixed in 4% paraformaldehyde for 20 min. After washing, the sample cells were permeabilised in the presence or absence of 0.5% Triton X-100 for 15 min. After another PBS wash, the sample cells were blocked with bovine serum albumin (BSA) for 30 min. The samples were incubated overnight at 4°C with primary antibodies: anti-LOX1 (HUABIO, ER1706-53, 1:50, China), anti-Caspase5 (HUABIO, ET1612-29, 1:50, China), and anti-CD206 (HUABIO, ET1702-04, 1:50, China). After washing off the unbound primary antibodies with PBS, the cells were incubated with fluorescent secondary antibodies, followed by nuclear staining and image acquisition. Lung tissue sections were stained with primary antibodies anti-iNOS (Servicebio, GB11119-100, 1:100, China) and anti-IL-10 (Servicebio, GB11534-100, 1:100, China). The following procedures were identical to those used for cellular immunofluorescence.

### 2.17 Statistical analysis

All statistical analyses were performed using Prism 8 or SPSS software, Statistical details are primarily provided in the figure legends. Data are presented as mean ± standard deviation. For comparisons involving three or more groups, analysis is performed using one-way ANOVA and Tukey’s multiple comparisons test; For comparisons between two groups, an unpaired parametric t-test is used. ****p < 0.0001, ***p < 0.001, **p < 0.01, *p < 0.05, not significant (ns), p > 0.05.

## 3 Results

### 3.1 Kp694 strain’s biological data and drug susceptibility

For Kp694, the minimum inhibitory concentrations (MICs) of MEM, tigecycline, and CA were 256, 2, and 10,000 μg/mL, respectively. For ATCC (700603), the MICs of MEM, tigecycline, and CA were 1, 0.5, and 10,000 μg/mL, respectively (Figure 1B). The clinical isolate Kp694 predicts coding genes based on sequencing results and uses the Comprehensive Antibiotic Resistance Database (CARD) and the Virulence Factor Database (VFDB) to predict and screen these genes. The classification results are shown in Figure 1C. The results of Gene Ontology (GO)/Kyoto Encyclopaedia of Genes and Genome (KEGG) annotations for protein-coding genes in the Kp694 strain are shown in Supplementary Figures S1A, B.

### 3.2 CA may alleviate Kp-MEM induced inflammation through OMV-dependent mechanism

The animal experiment procedure is depicted in Figure 1D, assuming that the high resistance of the clinical isolate Kp694 to MEM was unknown beforehand. To verify whether MEM aggravates infection through an OMV release-dependent factor, we used GSK-199, an orally administered OMV release inhibitor (Zhang et al., 2023), in combination with MEM. On the other hand, to preliminarily explore whether CA has protective effects, CA (40 mg/kg) was used in combination with MEM. The recorded mouse body weight results indicated a marked weight loss in the Kp + MEM group, while the Kp + MEM + GSK199 and Kp + MEM + CA groups exhibited a mitigated weight loss trend (Figure 1E). The expression levels of IL-6 and IL-1β in the mouse serum were assessed at 12, 24, and 48 h post-infection (Figures 1F, G). The results revealed that MEM treatment did not alleviate the inflammation levels in Kp-infected mice. On the contrary, serum IL-6 and IL-1β levels at 12 h were significantly higher in Kp + MEM mice than in Kp mice (P < 0.05). In contrast, serum IL-6 and IL-1β levels in the Kp + MEM + GSK199 group were lower than those in the Kp + MEM group at all three time points (P < 0.05). Similarly, serum IL-6 and IL-1β levels in the Kp + MEM + CA group were also reduced compared to those in the Kp + MEM group at 24 and 48 h (P < 0.05). The bacterial load results in lung, liver, and spleen tissues demonstrated that MEM treatment did not reduce Kp colonization in lung, liver, and spleen tissues (Figures 1H–J), and significantly increased bacterial loads in these tissues compared to the Kp group (P < 0.05). However, CA treatment reduced bacterial loads in these tissues (P < 0.05), while GSK199 did not show significant effects. These results first demonstrated that MEM treatment did not reduce Kp694 infection and colonization but instead intensified inflammation during the early stage of infection. Secondly, the combination of the OMV inhibitor and MEM effectively inhibited the release of pro-inflammatory factors but did not significantly inhibit bacterial colonization, supporting the hypothesis that KOMV-MEM released under MEM pressure is an important factor contributing to inflammatory damage. Finally, the combination of CA and MEM effectively reduced infection and colonization, suggesting that CA may alleviate inflammatory damage by inhibiting KOMV-MEM.

### 3.3 Impact of CA on OMV characteristics

**FIGURE 2:**
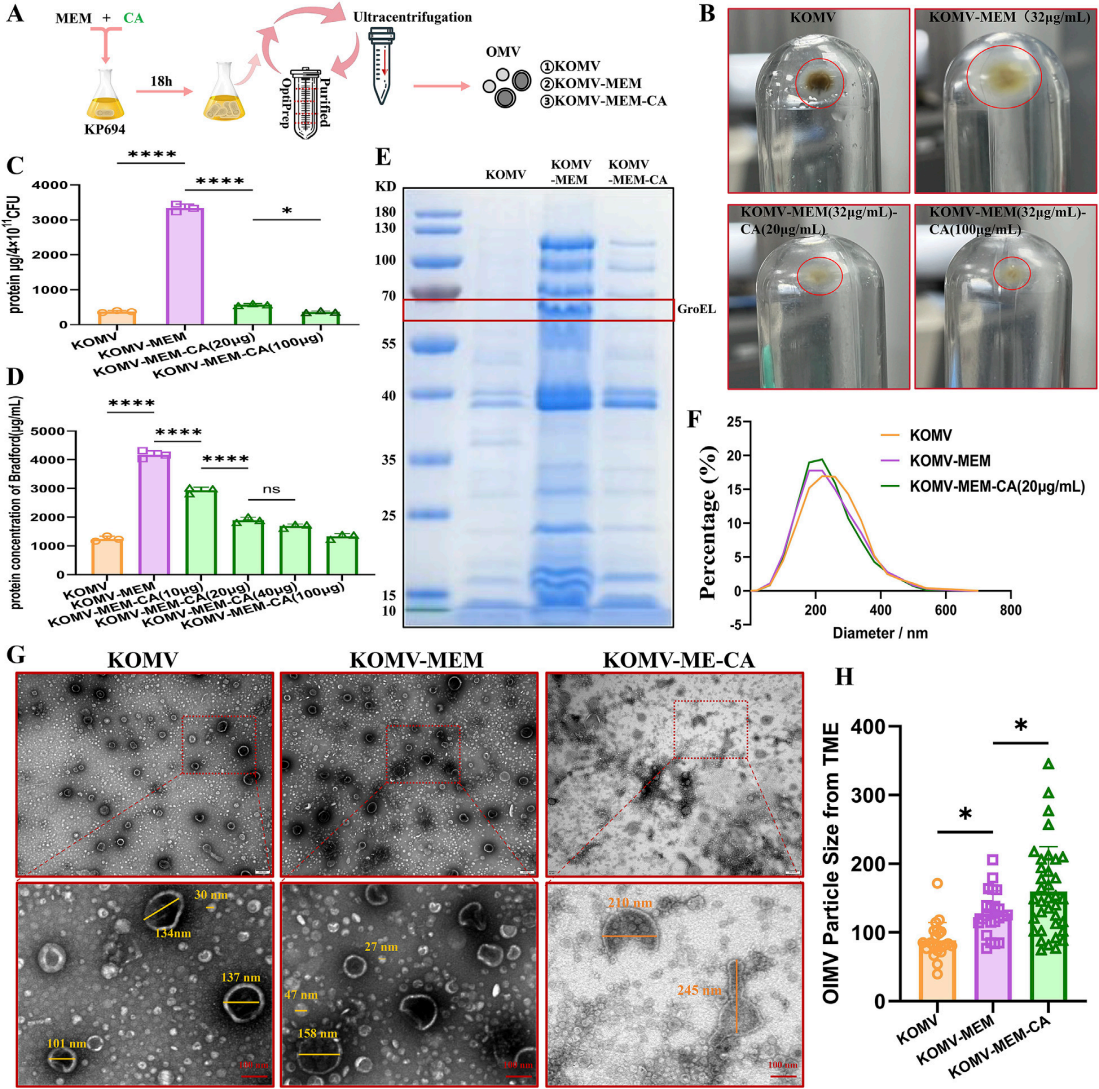
Impact of CA on the Characterization of OMV Release under MEM Pressure in Kp694. **(A)** Illustration of the OMV isolation and purification process. **(B)** Sedimentation of OMVs isolated and purified from groups with or without MEM and CA intervention under identical CFU conditions. **(C)** Total protein mass of OMVs collected per 4 × 10^11^ CFU Kp694 under conditions with or without MEM and CA intervention (n = 3). **(D)** Protein concentration of OMVs in each group at the same particle concentration (n = 3). **(E)** SDS-PAGE analysis of OMVs from each group at the same particle concentration. **(F)** Particle size and distribution of OMVs from each group at the same particle concentration detected by NTA (n = 4). **(G)** TEM observation of OMVs from each group at the same particle concentration in random fields of view (scale bars: 200/100 nm). **(H)** Average particle size of double-membraned vesicles (OIMVs) in OMVs from each group in random TEM fields of view, quantified using ImageJ software (n = 15). All data are expressed as mean ± S.D. *p < 0.05, ****p < 0.0001.

The vesicle collection and purification process is shown in Figure 2A. The effects of CA on KOMV-MEM were evaluated by comparing vesicle production, size, protein concentration, and structural characteristics. Compared to KOMV, KOMV-MEM production significantly increased, while CA intervention (KOMV-MEM-CA) reduced production (Figure 2B). Under the same CFU or particle concentration, Protein concentration was higher in KOMV-MEM than in KOMV (P < 0.0001), and reduced in KOMV-MEM-CA (P < 0.0001) (Figures 2C, D). No significant difference was observed between 20 and 40 μg/mL CA concentrations (P > 0.05) (Figure 2D). SDS-PAGE results showed a prominent 60 kDa protein band in KOMV-MEM (Figure 2E).

There is controversy over whether the size of OMVs increases or decreases under antibiotic stimulation compared to natural release (Ye et al., 2021; Zhang et al., 2023). Previous research has found that in *Helicobacter pylori*, larger OMVs contain a more diverse array of proteins compared to smaller OMVs (Turner et al., 2018). Li et al. (2024) found that most antibiotics induce *Escherichia coli* 47EC to secrete larger OMVs, and that the OMVs induced at 1/4 MIC are larger than those induced at 1/2 MIC, while tigecycline and meropenem induce the production of smaller OMVs. Furthermore, methodological differences in the isolation and measurement of OMVs may lead to varying results; for example, Dynamic Light Scattering (DLS) intensity weighting might overemphasize larger particles (Dehinwal et al., 2021). The single-membrane vesicles mostly have a size around 30 nm, and their protein and lipid content is too low, with a refractive index below the sensitivity limit of NTA, thus they cannot be detected (Bachurski et al., 2019). In view of this, the current study employed complementary NAT and TEM measurement methods to assess the size and integrity of KOMV-MEM under MEM conditions. NTA analysis revealed that KOMV-MEM had a smaller, more uniform size distribution than KOMV, while KOMV-MEM-CA had a more concentrated distribution (Figure 2F). TEM observations of double-membrane vesicles (OIMVs) loaded with more content (Figure 2G) indicate that their particle size mostly falls between 100 and 300 nm. Averaging only the OIMVs, results (Figure 2H) show a descending order of size from KOMV-MEM-CA to KOMV-MEM to KOMV. Therefore, we consider that antibiotic stress consistently pushes bacteria to offload components via vesiculation, however, the change in vesicle size depends on how bacteria balance packaging efficiency with membrane stability. It is noteworthy that CA intervention has impacted the structural integrity of these vesicle membranes, leading to irregular shapes and potential leakage, hence the particle size results might not reflect those loaded with more cargo.

### 3.4 CA inhibits KOMV-MEM induced lung injury

**FIGURE 3:**
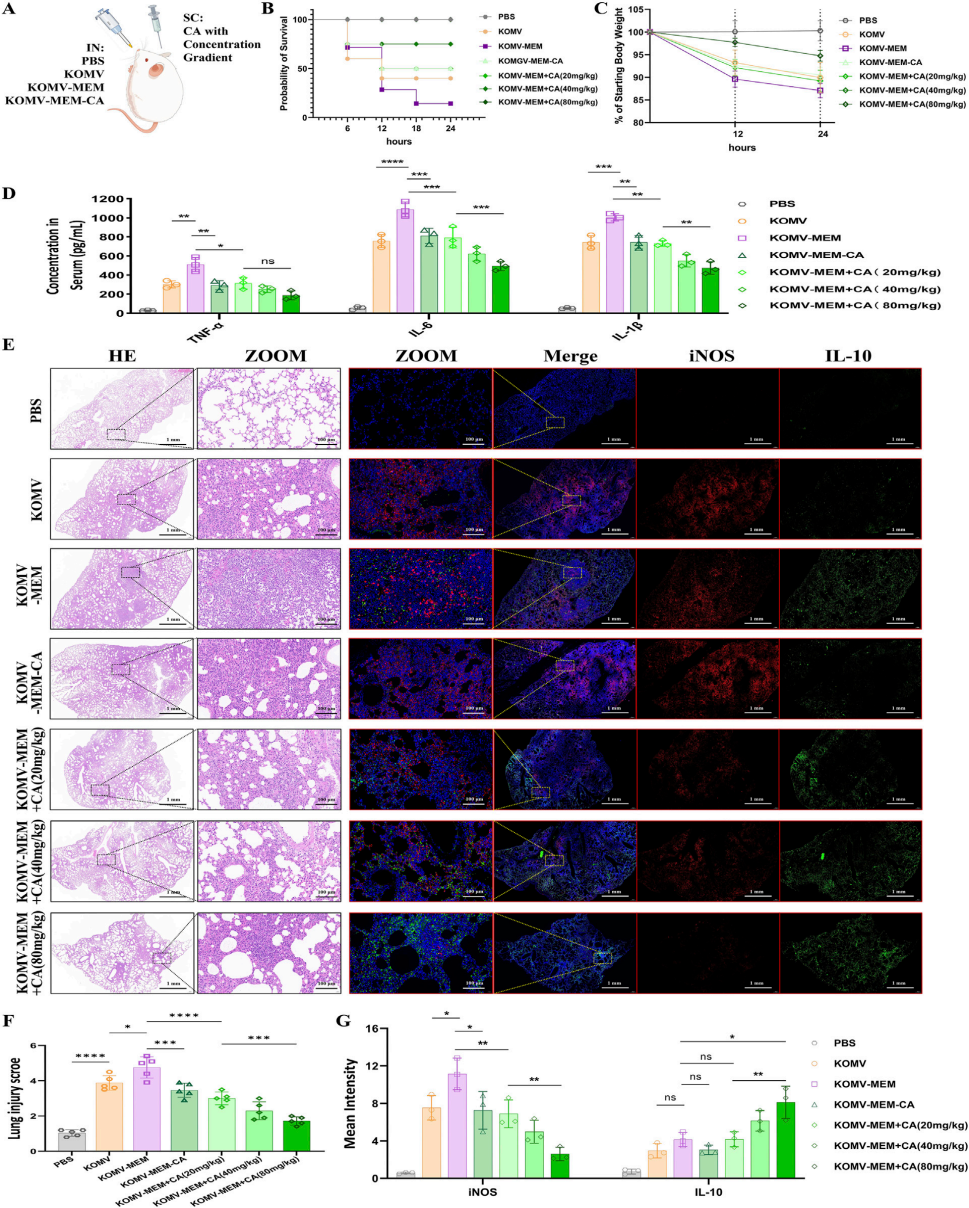
Impact of CA on KOMV-MEM-induced lung injury. **(A)** Animal experimental protocol. **(B)** Survival curve of experimental mice (n = 10). **(C)** Weight after infection (n = 10). **(D)** TNF-α, IL-6 and IL-1β concentration in the serum fluid, measured using ELISA (n = 3). **(E)** H&E staining images (scale bar: 1 mm or 100 µm)and immunofluorescence imaging (iNOS marked with red fluorescencescale, IL-10 marked with green fluorescencescale; bar: 1 mm or 100 µm) of consecutive lung tissue sections (n = 5). **(F)** Lung injury scores were determined using a seven-point scale ranging from 0 to 6, as follows: 0 indicates no injury, and six indicates extremely severe injury (n = 5). **(G)** Expression of iNOS and IL-10 quantified by average fluorescence intensity (n = 3). All data are expressed as mean ± S.D. *p < 0.05, **p < 0.01, ***p < 0.001, ****p < 0.0001.

Mice were intranasally administered with three types of vesicles and treated with CA at different concentrations (Figure 3A). After infection, compared with KOMV mice, KOMV-MEM mice exhibited the highest mortality, the most significant weight loss, and markedly elevated serum pro-inflammatory cytokine levels (TNF-α, IL-6, and IL-1β) (P < 0.01); In contrast, compared to KOMV-MEM mice, KOMV-MEM-CA mice exhibited lower mortality and a reduced trend in weight loss, along with a significant decrease in serum pro-inflammatory cytokine levels (P < 0.01). These findings suggest that KOMV-MEM infection induces a strong inflammatory response, whereas the virulence of KOMV-MEM-CA is attenuated. Furthermore, mice receiving subcutaneous CA treatment (KOMV-MEM + CA) also exhibited reduced mortality and lower levels of inflammation, with the 80 mg/kg dose being more effective than the 20 mg/kg dose in lowering IL-6 and IL-1β levels (P < 0.01) (Figures 3B–D). These findings confirm that CA reduces inflammation induced by KOMV-MEM. To further investigate the effects of CA on lung tissue damage and immune environment in KOMV-MEM infected mice, we performed HE staining and immunofluorescence quantitative analysis of macrophage M1 and M2 polarization markers iNOS and IL-10 on two consecutive lung tissue sections (Figure 3E). Histopathological staining results indicate that, compared with KOMV, the lung tissues of KOMV-MEM mice exhibit a larger area of consolidation and more severe inflammatory infiltration around the bronchi. The pathological changes in the lung tissues of KOMV-MEM-CA/+CA mice were relatively milder, and the reduction in lung pathology in +CA mice was dose-dependent. Inflammatory scores (0–6) were assigned to each group based on lung histological changes (Figure 3F). Since HE staining and immunofluorescence were carried out on consecutive sections, a comparison of the overlapping areas showed that in the severely affected regions of the KOMV and KOMV-MEM groups, iNOS expression was markedly elevated. Notably, KOMV-MEM mice exhibited greater iNOS activation (P > 0.05), which may be attributed to the retention of inflammatory regions and extensive M1 polarization of recruited macrophages. On the other hand, compared with KOMV-MEM mice, the KOMV-MEM-CA/+CA groups showed a significant reduction in iNOS levels (P < 0.05), with a statistically significant difference between the 80 mg/kg and 20 mg/kg groups (P < 0.01), indicating a more pronounced inhibition of M1 polarization (Figure 3G). IL-10 expression did not differ significantly among groups, but was higher in CA (80 mg/kg) treated mice compared to the KOMV-MEM group (P < 0.05) (Figure 3G). These results demonstrate that KOMV-MEM causes more severe lung infection, while CA exerts immunomodulatory effects to alleviate inflammation.

### 3.5 CA regulates the KOMV-MEM proteome

**FIGURE 4:**
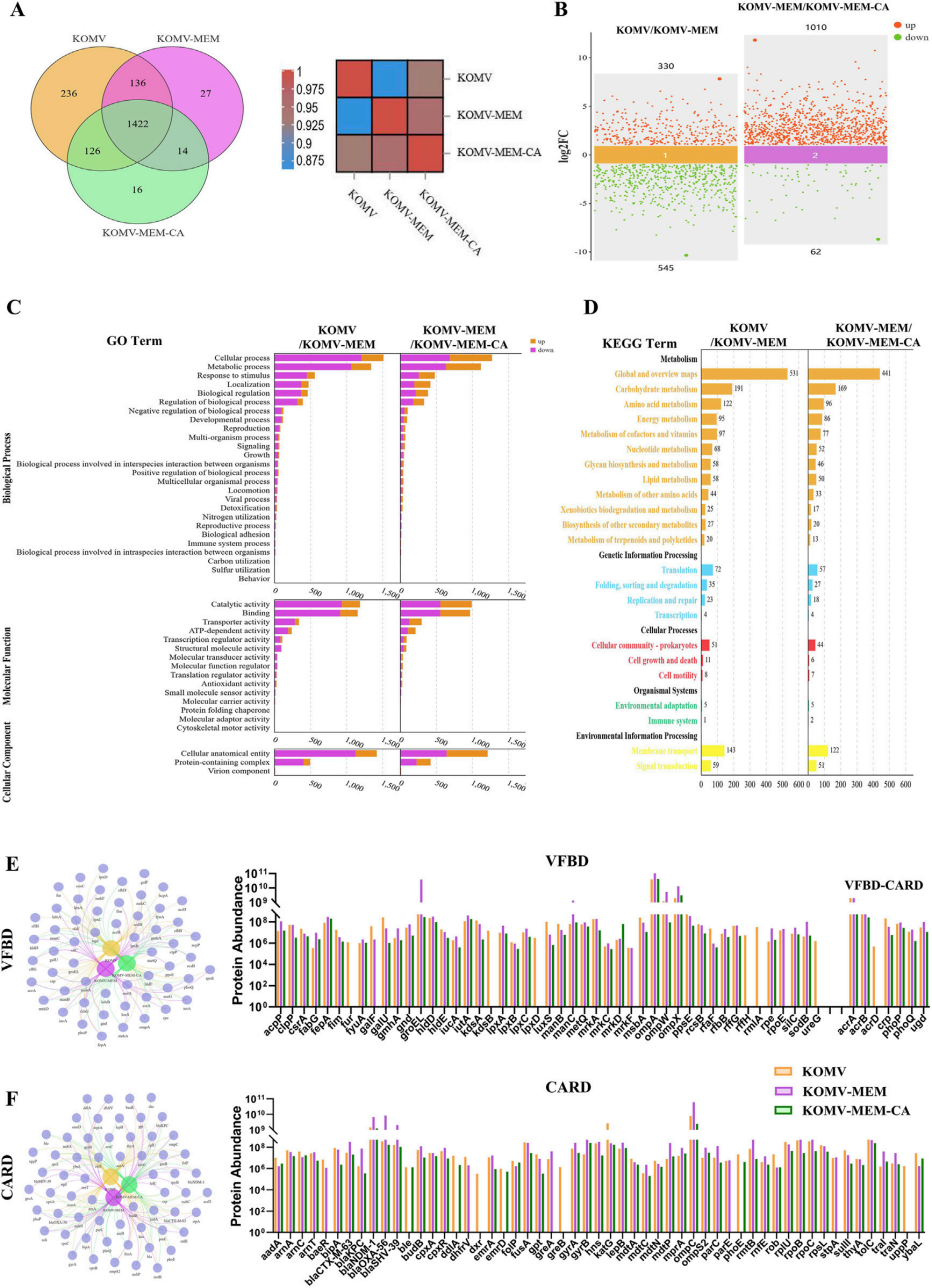
Proteomic analysis of KOMV, KOMV-MEM, and KOMV-MEM-CA. **(A)** Protein relationships between groups (Venn diagram and heatmap). **(B)** Scatter plot of protein differences between groups. **(C, D)** GO/KEGG analysis of protein differences between groups. **(E, F)** VFBD and CARD annotation results of samples. Relevant protein network generation - Venn diagram and abundance difference bar chart.

Proteomic analysis at the same OMV particle concentration identified 1920, 1599, and 1578 differential representative proteins in KOMV, KOMV-MEM, and KOMV-MEM-CA, respectively. Intergroup correlation analysis as shown in Figure 4A indicates significant protein type differences between KOMV and KOMV-MEM, while the proteome of KOMV-MEM-CA is more similar to KOMV-MEM. Comparative analysis (Figure 4B) shows that KOMV has 330 upregulated and 545 downregulated proteins compared to KOMV-MEM; KOMV-MEM has 1010 upregulated and 62 downregulated proteins compared to KOMV-MEM-CA. GO and KEGG analyses demonstrated distinct functional profiles among KOMV, KOMV-MEM, and KOMV-MEM-CA. GO analysis revealed (Figure 4C) significant differences between KOMV and KOMV-MEM in biological processes, with KOMV-MEM showing upregulated proteins related to metabolic processes, stimulus response (biological processes), catalytic activity/binding (molecular functions), and cellular components like protein complexes. KOMV-MEM-CA showed similar regulation patterns to KOMV-MEM in these processes. KEGG pathway enrichment highlighted differential protein clustering in metabolic pathways (carbohydrate/amino acid metabolism), genetic information processing (translation, protein folding), and environmental response (membrane transport) (Figure 4D). MEM modification enhanced OMVs’ metabolic regulation and cellular signaling capabilities. MEM-CA intervention modulated carbohydrate/amino acid metabolism and membrane transport pathways, potentially influencing energy metabolism and protein functionality. These results collectively indicate that MEM and MEM-CA interventions critically reshape OMVs’ proteomic composition, particularly affecting metabolic coordination, protein synthesis, and membrane dynamics. Using VFBD and CARD databases to annotate proteins, the classified protein relationship networks and intergroup abundance differences are shown in Figures 4E, F (using gene symbols), with an increased abundance of virulence-related proteins in KOMV-MEM compared to KOMV, including extracellular factors such as SodB (superoxide dismutase), HldD (lipopolysaccharide synthase); iron acquisition protein FyuA (Ferric yersiniabactin uptake protein A); adhesion-related factors such as OmpA (outer membrane protein A), OmpW, OmpX. Antibiotic pressure can trigger a stress response in bacteria, leading to increased expression of certain virulence factors to help them survive adverse conditions. For example, studies have found that in *Acinetobacter* baumannii, treatment with carbapenems (such as meropenem) significantly increases the mRNA levels of outer membrane proteins OmpA and OmpW in persister cells. The upregulation of these outer membrane proteins is believed to help maintain outer membrane function and integrity in the presence of high concentrations of antibiotics, thereby enhancing survival (Schmitt et al., 2023). Additionally, previous SDS-PAGE results suggested that the protein with significantly elevated expression at around 60 kDa in KOMV-MEM was validated through proteomic analysis as GroEL chaperonin, confirming its increased abundance in KOMV-MEM. GroEL is a heat-shock chaperone protein that assists in folding nascent polypeptide chains and prevents protein misfolding. Interestingly, GroEL can also perform nontraditional functions on the bacterial surface, such as mediating adhesion to host fibronectin/adhesins and binding iron ions (Fourie and Wilson, 2020). Relative to KOMV-MEM, the abundance of these proteins was reduced in the KOMV-MEM-CA group. The proteins encoded by related resistance genes mainly involve mechanisms such as β-lactam, Fluoroquinolone resistance, Aminoglycoside resistance, Multidrug Efflux Pumps, Antibiotic Target Modification and Protection. Compared to KOMV, the abundance of β-lactam (blaKPC, blaCTX-M-63, blaSHV-39, blaOXA-56, blaNDM-1), Fluoroquinolone (GyrA, GyrB, ParC, ParE), Aminoglycoside (RmtB), and Multidrug Efflux Pumps (MdtA, MdtC, MdtN, MdtP) as well as associated regulators was upregulated in KOMV-MEM, while these proteins were downregulated in KOMV-MEM-CA compared to KOMV-MEM. Furthermore, proteins related to T4SS-mediated transfer of the F plasmid, TraI and TraN, were upregulated in KOMV-MEM but decreased after CA intervention. This suggests that CA may interfere with the proteins highly expressed under MEM stimulation by inhibiting the regulatory pathways that mediate virulence and resistance gene expression, while CA may also exhibit membrane-stabilizing effects that alleviate bacterial stress; however, the precise mechanism requires further investigation.

### 3.6 CA mitigates KOMV-mem-induced macrophage pyroptosis by inhibiting GroEL-LOX1 expression

**FIGURE 5:**
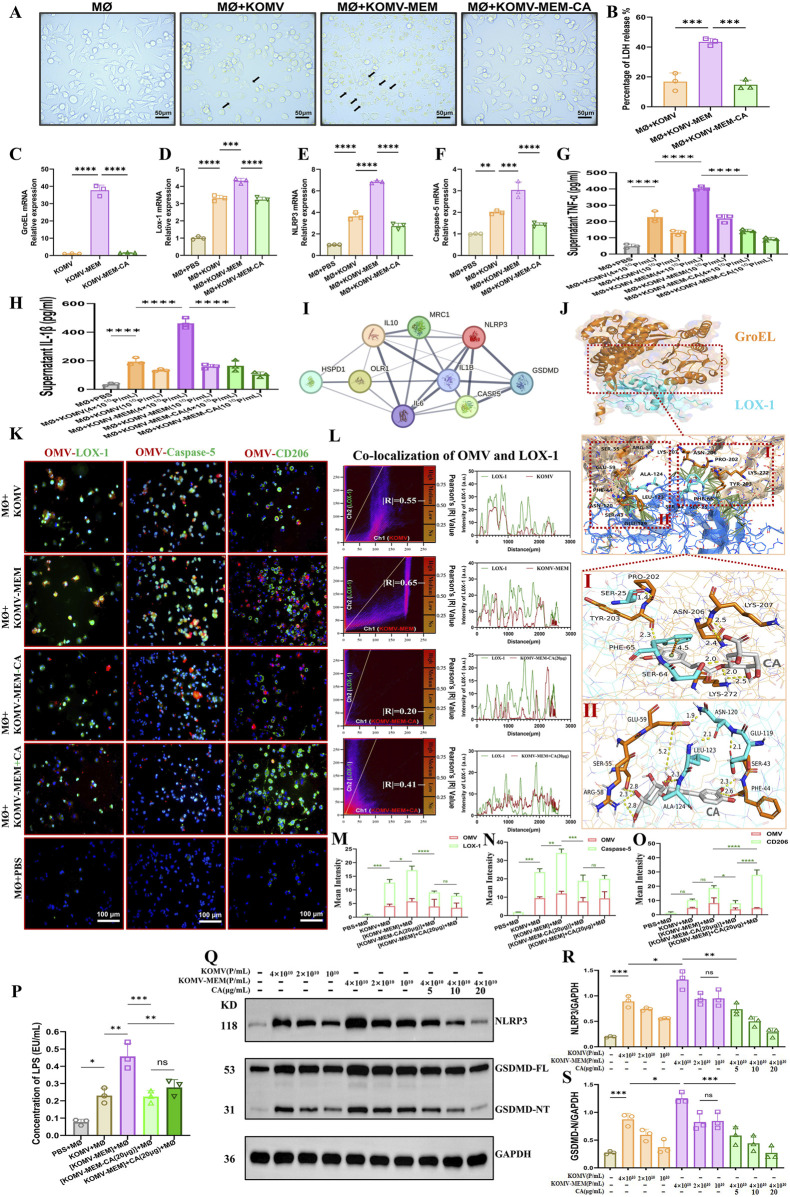
CA Inhibits Pyroptosis Induced by KOMV-MEM in Macrophages. **(A)** Cell morphology observed under an optical microscope 6 h after stimulation with KOMV, KOMV-MEM, and KOMV-MEM-CA; arrows indicate pyroptosis characteristics (particle concentration for each group was 1 × 10^10^ Particles/mL, scale bar 50 μm). **(B)** LDH release detected by LDH cytotoxicity assay kit (particle concentration for each group was 10^10^ Particles/mL, n = 3). **(C)** PCR detection of GroEL mRNA expression in the KOMV, KOMV-MEM and KOMV-MEM-CA (n = 3). **(D–F)** RNA extracted from macrophages 6 h after stimulation, reverse transcribed, and PCR used to detect mRNA expression levels of LOX-1, NLRP3, and Caspase-5 (n = 3). **(G, H)** ELISA kits used to measure levels of inflammatory cytokines TNF-α, IL-1β in cell supernatants (particle concentration for each group was 1–4 × 10^10^ Particles/mL, n = 3). **(I)** PPI of GroEL and LOX-1 **(J)** Prediction of GroEL (PDB ID: 8BL2) and LOX-1 (PDB ID: 7W5D) Protein Interaction Using Alphafold 3. **(K)** Macrophages imaged under a fluorescence microscope after co-incubation with KOMV, KOMV-MEM, KOMV-MEM-CA, KOMV-MEM + CA; OMVs marked with red fluorescence, macrophages labeled through green fluorescent secondary antibodies binding to primary antibodies against LOX-1, Caspase-5, CD206, and macrophage nuclei marked with DAPI blue fluorescence (scale bar 50 μm). **(L)** Colocalization images of cells and OMVs, along with Pearson correlation coefficient **(M–O)** Expression of OMV, LOX-1, Caspase-5, CD206 quantified by average fluorescence intensity (n = 3). **(P)** LAL endotoxin assay for cytoplasmic LPS (particle concentration for each group was 4 × 10^10^ Particles/mL, n = 3) **(Q–S)** Protein expression levels of NLRP3 and GSDMD-NT in cells under different intervention conditions. All data are expressed as mean ± S.D. *p < 0.05, **p < 0.01, ***p < 0.001, ****p < 0.0001.

Antibiotic stress results in the accumulation of misfolded and toxic proteins in the bacterial periplasm, thereby enhancing the virulence of vesicular membrane proteins (McBroom and Kuehn, 2007). With identical particle concentrations, interventions in MØ cells were performed using three kinds of vesicles: KOMV, KOMV-MEM, and KOMV-MEM-CA. The results indicated that KOMV-MEM triggered a more severe pyroptosis than KOMV, as evidenced by the secretion of active lysosomes, cell swelling, and lysis. KOMV-MEM-CA induced less pyroptosis (Figure 5A) with a significantly (P < 0.001) lower percentage of lactate dehydrogenase (LDH) release than MØ+KOMV-MEM (Figure 5B). GroEL can promotes the refolding of misfolded proteins (Omnus et al., 2023). Previous research has shown that antibiotic pressure causes a significant accumulation of the membrane protein, GroEL, in OMVs released by *K. pneumoniae*, which is specifically recognised by the macrophage membrane receptor LOX-1, leading to substantial uptake of OMVs by macrophages (Ye et al., 2021). According to CCK-8 results, the highest non-toxic concentration of CA in M0 cells is 20 μg/mL. Therefore, we will use 20 μg/mL as the maximum concentration in our cell experiments (Supplementary Figure S2). PCR results showed a significant increase in GroEL mRNA levels in KOMV-MEM, whereas there was almost no expression in KOMV and KOMV-MEM-CA (Figure 5C), this is consistent with the trend of the results identified in the proteome (Figure 4E). Similarly, under different intervention conditions, the expression of the macrophage LOX-1 mRNA in the MØ+KOMV-MEM-CA group was lower than that in the MØ+KOMV and MØ+KOMV-MEM groups (Figure 5D). LPS can trigger atypical pyroptosis via Caspase-5 (human-origin) (Vanaja et al., 2016). Following the substantial recognition and uptake of OMVs and LPS by macrophages via the LOX-1 receptor, NLRP3 and Caspase-5 are directly activated. Caspase-5 induces cell pyroptosis by cleaving gasdermin D and indirectly mediates the maturation of interleukin (IL)-1β through the NLRP3-ASC inflammasome, triggering intense inflammation (Choi et al., 2019). PCR results indicate that compared with the MØ+KOMV-MEM group, NLRP3 and Caspase-5 mRNA levels decreased in the MØ+KOMV-MEM-CA group (Figures 5E, F). Pro-inflammatory cytokines TNF-α and IL-1β were measured in cell supernatants, showing significantly higher levels in MØ+KOMV-MEM than in other groups (P < 0.0001) (Figures 5G, H). The Protein-Protein Interaction (PPI) between GroEL and LOX-1 is illustrated in Figure 5I. The interaction mechanism between GroEL and LOX-1 was predicted using Alphafold 3, and CA was docked with the interacting proteins to predict potential intervention targets. AlphaFold 3 predicted a confident interaction between GroEL and LOX-1, with a confidence score (pLDDT) between 70 and 90 for the interacting regions (Figure 5J), indicating the presence of a stable interaction interface. The primary contact surface features interactions between GroEL residues SER-43, ASN-120, PHE-44, GLU-59, SER-55, ARG-58, LYS-207, ASN-206, PRO-202, LYS-272, TYR-203 and LOX-1 residues LEU-123, ALA-124, SER-64, SER-25, PHE-65. These interactions suggest a strong electrostatic complementarity between the two proteins. The forecasted interaction between GroEL and LOX-1 implies a functional partnership in cellular signaling pathways, with potential intervention targets for CA predicted via molecular docking. CA has two docking pockets (Ⅰ and Ⅱ) at the interaction interface, with binding energies of 9.6 and 7.5 KCAL/MOL, respectively. CA has two docking pockets (Ⅰ and Ⅱ) at the interaction interface, with binding energies of 9.6 and 7.5 KCAL/MOL, respectively. Ⅰ: CA is fixed with GroEL residues TYR-203, ASN-206, LYS-202 through hydrogen bonds, and with LOX-1 residue PHE-65 through hydrogen bonds, and binds with residue SER-64 through π-π stacking interactions. Ⅱ: CA is anchored to GroEL residues SER-43, PHE-44, GLU-59, SER-55, ARG-58 via hydrogen bonds, and to LOX-1 residue ALA-124 via hydrogen bonds.

Each group’s OMVs were marked with a red fluorescent dye, Dil, and then introduced to the MØ cells. Fluorescence analysis showed increased LOX-1 and Caspase-5 expression and colocalization with OMVs in the KOMV-MEM group. In KOMV-MEM-CA and KOMV-MEM + CA groups, LOX-1 and Caspase-5 expression decreased, with reduced OMV association, and there was no significant difference in pyroptosis levels between -CA and +CA groups (Figures 5K–N). This indicates that CA might directly modulate MØ to foster an anti-inflammatory response, as evidenced by enhanced CD206 expression in the MØ+CA group (P < 0.0001) compared to KOMV-MEM-CA (Figure 5O). The cytoplasmic LPS content analysis (Figure 5P) showed that under the same particle concentration, cells in the KOMV-MEM group took up the most LPS, with a significant statistical difference compared to the -CA and +CA groups (P < 0.01). This is consistent with the trend observed in the co-localization results between cells and OMVs. Finally, we intervened MØ with different particle concentrations of KOMV and KOMV-MEM, and then added different concentrations of CA. Subsequently, we detected the expression of NLRP3 and GSDMD-NT proteins in cells to assess the extent of pyroptosis. As shown in Figures 5Q–S, CA significantly inhibited pyroptosis in a dose-dependent manner, with the most notable inhibition at 20 μg/mL (P < 0.05).

### 3.7 CA inhibits the horizontal transfer of resistance genes in KOMV-MEM

**FIGURE 6:**
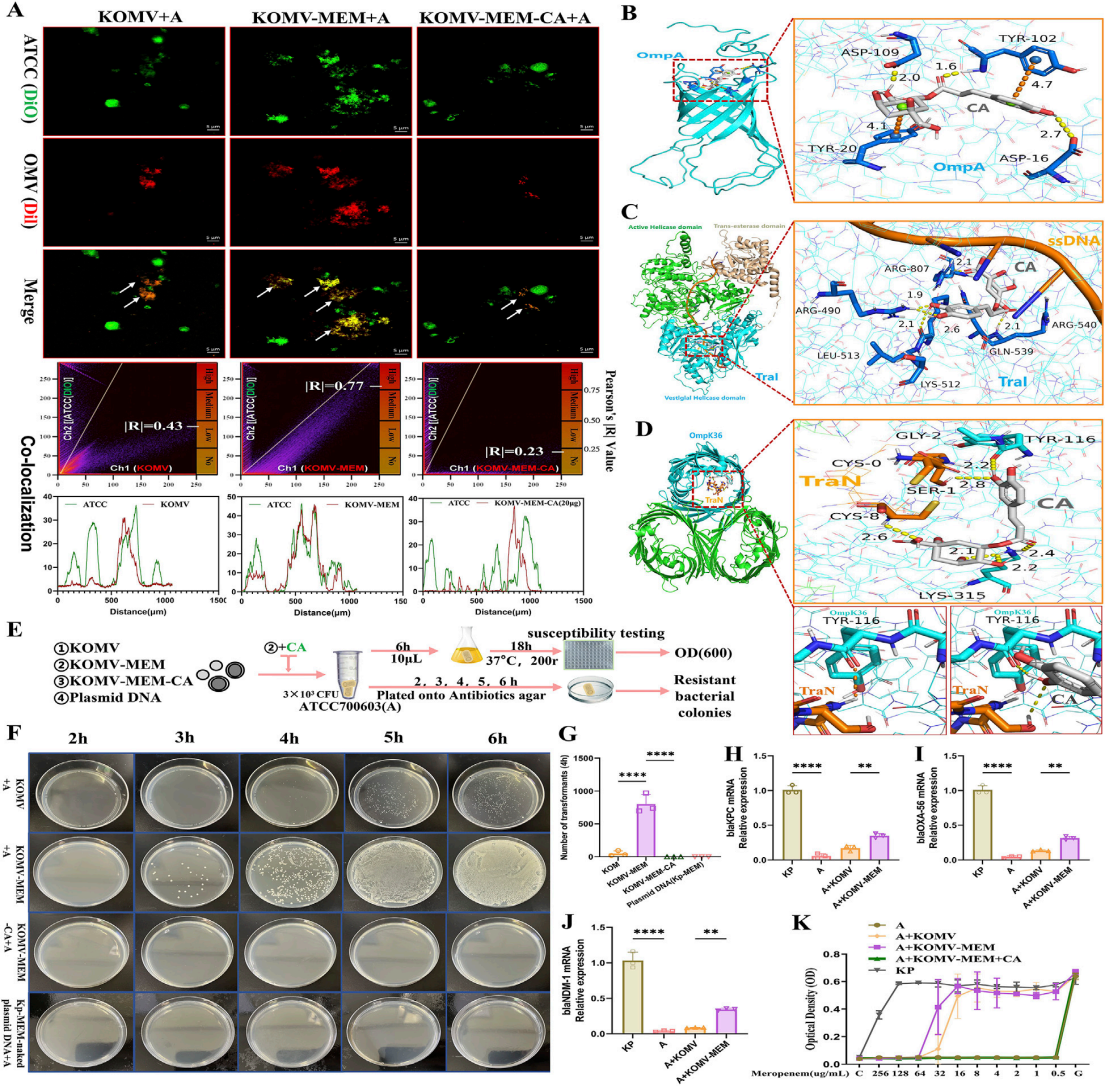
CA Intervention in KOMV-MEM Mediated Transfer of Antibiotic Resistance Genes. **(A)** ATCC 700603 bacteria alongside KOMV, KOMV-MEM, and KOMV-MEM-CA visualized under confocal microscopy (scale bar: 5 μm), with OMVs depicted in Channel 1 (red fluorescence) and ATCC 700603 in Channel 2 (green fluorescence). Shows colocalization images alongside Pearson correlation coefficients. **(B–D)** Molecular docking imagery demonstrates the binding posture and interactions between CA (white skeleton, PubChem CID: 1794427) and OmpA (PDB ID: 2K0L), TraI-ssDNA (PDB ID:5N8O), TraN-OmpK36(PDB ID:7SZI), where yellow dashed lines indicate hydrogen bond forces and orange dashed lines signify π-π stacking forces. **(E)** Illustrates the experimental design and workflow for CA intervention in the transfer of antibiotic resistance genes by KOMV-MEM. **(F, G)** ATCC 700603 was co-incubated with KOMV, KOMV-MEM, KOMV-MEM-CA, and Kp-MEM naked plasmid for 2–6 h. Every hour, 100 μL of the bacterial solution was plated on agar plates containing MEM (32 μg/mL), photographed, and counted. **(H–J)** After co-incubation for 6 h, resistant colonies were collected for PCR to detect the expression levels of resistance genes (blaKPC, blaOXA-56, blaNDM-1) (n = 3). **(K)** After 6 h of co-incubation, 10 μL of bacterial solution was cultured in fresh LB medium for 18 h, and 1 × 10^5^ CFU/mL bacterial solution was used to test MEM susceptibility (OD600) in the presence or absence of CA (n = 5). All data are expressed as mean ± S.D. **p < 0.01, ****p < 0.0001.

Horizontal gene transfer (HGT) between bacteria is a complex biological process in which OMVs play an important role, serving as mediators of cargo transportation between microorganisms and facilitating the transmission of antibiotic resistance genes (Li et al., 2022). To investigate the impact of CA on OMV-strain conjugation, we labelled the susceptible strain ATCC (700603) with a green fluorescent dye (DIO). After ensuring that KOMV, KOMV-MEM, and KOMV-MEM-CA have equal particle concentrations, they were labelled with a red fluorescent dye (DIL). Following thorough mixing, the mixture was co-incubated for 4 h and imaged using laser confocal microscopy for colocalization analysis of the vesicles and strains. As shown in Figure 6A, KOMV-MEM conjugated more with the bacterial strain, with a Pearson’s R-value of 0.77, indicating a significant overlap of the two fluorescence signals in the peak chart. Conversely, KOMV-MEM-CA exhibited the lowest conjugation with a Pearson R-value of only 0.23.

In addition to the pathways through which OMVs internalize cargo into the cytoplasm via proximal lysis or fusion to transfer antibiotic resistance genes (Fulsundar et al., 2014), we predict that the F-plasmid transfer mechanism mediated by T4SS may also be involved (Macé et al., 2022). Based on the proteome (Figure 4F), we screened three proteins that are highly associated with the transfer of antibiotic resistance genes: ompA, traI, and traN, and simulated potential intervention sites of CA at the molecular level. OmpA can facilitate adhesion and conjugation (Ozkanca et al., 2002). The optimal binding pose of CA with OmpA is shown in Figure 6B, with a binding energy of 7.4 KCAL/MOL, tightly fixed with residues ASP-109, TYR-102, and ASP-16 through hydrogen bonds, and bound to residues TYR-102 and TYR-20 through π-π stacking interactions. This model structure characterizes the molecular basis of CA’s effect on OmpA bioactivity. TraI is an essential protein for the conjugation process driving bacterial adaptation and evolution and is a primary driving force for the spread of antibiotic resistance genes (Macé et al., 2022). The TraI-ssDNA complex model was used to simulate the unwinding process of resistant plasmids before entering recipient bacteria, and molecular docking was used to simulate the potential binding sites of CA. The results showed (Figure 6C) that the optimal binding site of CA with TraI is located at the vestigial helicase domain, with a binding energy of 9.1 KCAL/MOL. This domain is a key site for plasmid DNA unwinding (Ilangovan et al., 2017). CA disrupted the contact surface between ssDNA and residue ARG-540. CA was fixed with residues ARG-807, ARG-490, LYS-512, and GLN-539 through hydrogen bonds. Studies have identified the importance of OM “pairing stability” mediated by *K. pneumoniae* TraN-OmpK36 in driving efficient plasmid transfer, and constructed the crystal structure of the TraN-OmpK36 complex (Low et al., 2022). We retained the two protein complex structures (TraN-OmpK36) to simulate the efficient plasmid delivery process of KOMV. Molecular docking was used to confirm the optimal binding site of CA with TraN-OmpK36 (Figure 6D), with a binding energy of 7.9 KCAL/MOL. CA was fixed to TraN residues GLY-2 and CYS-8, and OmpK36 residues TYR-116 and LYS-315 through hydrogen bonds. After docking, CA disrupted the hydrogen bond interaction between TYR-116 and TraN. These results predict potential targets for CA to inhibit OMV-mediated antibiotic resistance plasmid transfer.

To further investigate the effect of CA on KOMV-MEM antibiotic resistance gene transfer, three types of vesicles at the same particle concentration (1 × 10^11^ Particles/mL) and Kp-MEM-naked plasmid were co-cultured with log phase ATCC700603 strain (A) at 37°C. At 2, 3, 4, 5, and 6 h time points post-culture, equal amounts of bacterial solution from each group were plated on agar plates containing MEM (Figure 6E). The results showed (Figure 6F) that KOMV + A obtained resistant colonies at 4 h, KOMV-MEM + A obtained resistant colonies at 3 h. However, no resistant colonies were obtained in the KOMV-MEM-CA + A and plasmid + A natural transformation groups. The number of colony transformations was calculated at the 4 h time point (Figure 6G). PCR identification results of resistant colonies collected at 6 h showed (Figures 6H–J) that KOMV + A and KOMV-MEM + A acquired blaKPC, blaOXA-56, and blaNDM-1 antibiotic resistance genes to varying degrees, with KOMV-MEM having a higher transfer efficiency than KOMV (P < 0.01). On the other hand, to study the direct effect of CA on the gene transfer of KOMV-MEM to recipient bacteria, free CA was added during the co-culture of the collected KOMV-MEM with the sensitive strain (distinct from KOMV-MEM-CA). After co-incubation for 6h, 10 μL was taken and re-shaken in fresh LB for 18 h to ensure genetic stability of the bacterial population. Then MEM sensitivity tests were conducted for each group. The results, as shown in Figure 6K, indicated that KOMV + A and KOMV-MEM + A were still resistant to MEM to varying degrees, while KOMV-MEM + CA + A was sensitive to MEM and did not acquire resistance.

### 3.8 CA inhibits KOMV-MEM mediated antibiotic hydrolysis thereby reducing bacterial colonization

**FIGURE 7:**
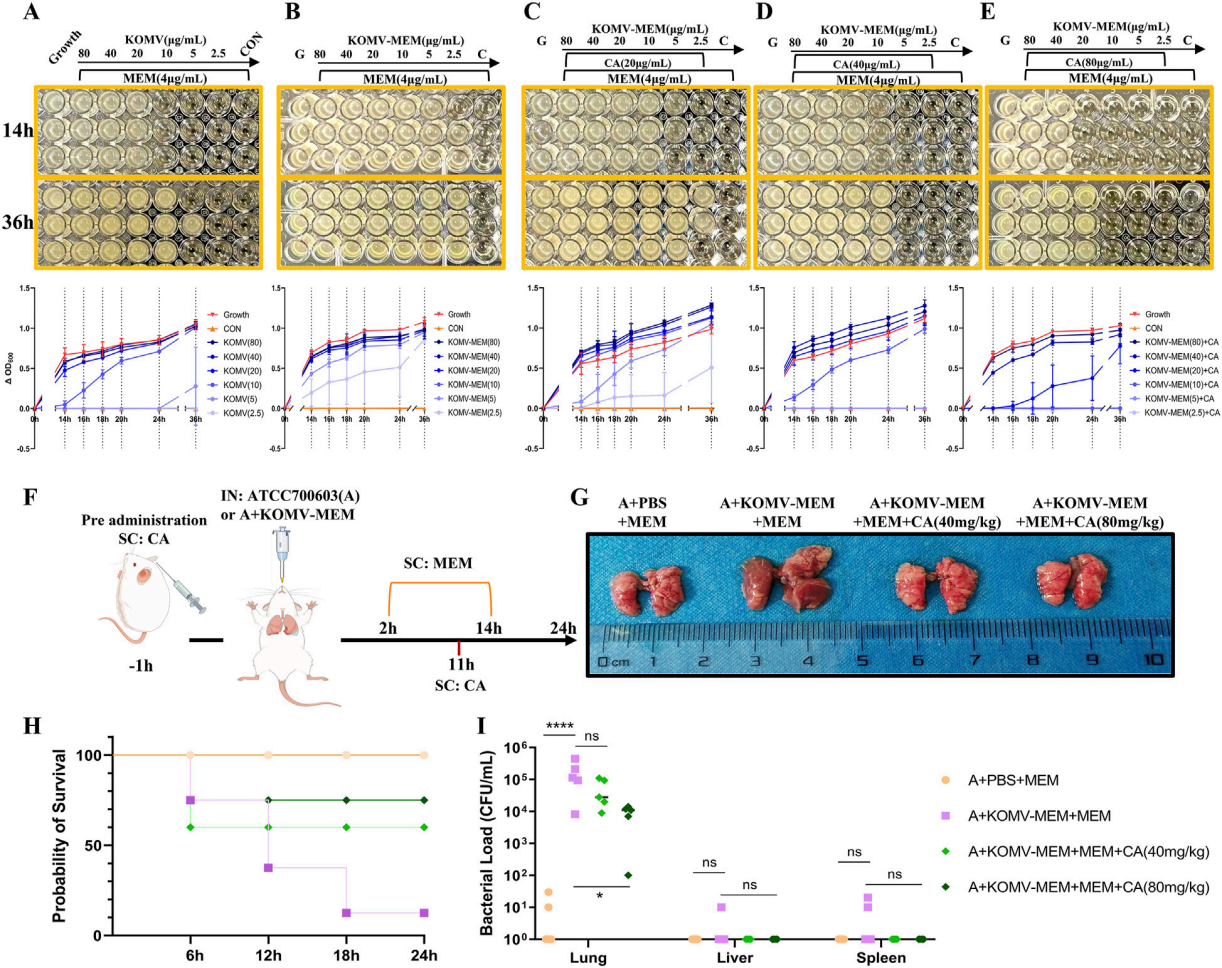
CA inhibits KOMV-MEM mediated antibiotic hydrolysis. **(A–E)** CA inhibits KOMV-MEM mediated meropenem hydrolysis to protect susceptible bacterial strains. The growth of ATCC 700603 was monitored under different conditions at 4 μg/mL MEM antibiotic concentration, and dynamic growth curves of the bacteria were plotted over 14–36 h (n = 3). **(F)** Experimental protocol for *in vivo* study of CA inhibition on KOMV-MEM mediated antibiotic hydrolysis. **(G)** Morphology of mouse lungs. **(H)** Survival curve of experimental mice (n = 10). **(I)** Bacterial load in Lungs, Liver and Spleen (n = 5). All data are expressed as mean ± S.D. *p < 0.05, ****p < 0.0001.

Bacteria or OMVs hydrolyze antibiotics depending on the antibiotic-inactivating enzymes they carry, such as *K. pneumoniae* carbapenemase and New Delhi Metallo-beta-lactamase (Martínez et al., 2021; Saeki and Sasaki, 2023). We hypothesized that CA could degrade the carbapenemase carried by KOMV-MEM. KOMV and KOMV-MEM were adjusted to similar protein concentrations and diluted to six concentrations (2.5, 5, 10, 20, 40, 80 μg/mL). CA was added at 20, 40, and 80 μg/mL, along with logarithmic-phase ATCC700603 (10^5^ CFU/mL) and MEM (4 μg/mL, 4×MIC). OD600 readings revealed that KOMV exhibited MEM hydrolytic activity, with 5 μg of protein consuming 4 μg of MEM (Figure 7A). KOMV-MEM showed stronger hydrolytic activity, completely hydrolyzing 4 μg of MEM at protein concentrations below 2.5 μg (Figure 7B). CA intervention significantly inhibited the ability of KOMV-MEM to hydrolyze antibiotics, and the inhibitory effect on KOMV-MEM increased with higher CA concentrations (Figures 7C-E). When 40 μg and 80 μg of CA were added to KOMV-MEM with protein amounts of 5 μg and 10 μg, respectively, KOMV-MEM completely lost its ability to protect susceptible bacteria. *In vivo*, susceptible bacteria and KOMV-MEM were administered intranasally to mice, followed by subcutaneous MEM injections and CA at 40 and 80 mg/kg (Figure 7F). At 24 h post-inoculation, CA treatment improved lung morphology, reduced congestion, edema, and hemorrhagic spots, and decreased mortality, especially at the 80 mg/kg dose (Figures 7G, H). Since the inoculated bacteria were sensitive to MEM, only a minimal amount of bacteria was detected in the lung tissues of PBS + MEM mice, whereas the bacterial load was significantly increased in the lung tissues of KOMV-MEM + MEM mice (P < 0.0001). Bacterial loads in the lung tissues of the CA (80 mg/kg) group were reduced compared to those in the KOMV-MEM + MEM mice (P < 0.05) (Figure 7I). The results confirmed that KOMV-MEM can protect susceptible bacterial strains *in vivo* by hydrolyzing MEM, and that CA inhibits the antibiotic-inactivating enzymes carried by KOMV-MEM.

## 4 Discussion

The threat of MDR bacteria is a global public health concern, as antibiotic misuse has led to the evolution of bacterial resistance (Upadhayay et al., 2023). In response to antibiotic pressure, bacteria release OMVs, which can facilitate the transfer of resistance to enhance adaptability (Saeki and Sasaki, 2023). Furthermore, OMVs released under antibiotic pressure pose a greater threat to the immune system (Ye et al., 2021). In this study, we established a mouse model of intranasal infection with Kp694 (MEM-resistant) and found that MEM treatment did not reduce bacterial colonization in mouse tissues, but rather induced stronger inflammation at the early stage of infection. The combination of GSK-199 (an OMVs release inhibitor) and MEM reduced the release of pro-inflammatory factors IL-6 and IL-1β, but did not significantly inhibit bacterial colonization. The inhibition of inflammation without a reduction in bacterial load indirectly suggests that MEM-induced inflammatory exacerbation is OMV-mediated. Interestingly, the combination of MEM and CA demonstrated a similarly significant anti-infective effect. In our *in vitro* susceptibility tests, the MIC of CA against Kp was approximately 10 mg/mL, which is consistent with previous reports (Tan et al., 2020), but this is an unachievable plasma concentration in humans (Kumar et al., 2019). Therefore, we propose for the first time that the inhibition of OMVs-mediated virulence and resistance transmission by CA may be an important antibacterial mechanism.

This study confirmed that CA inhibited the quantity, structure, and proteins of OMVs released by Kp under MEM pressure. Antibiotic pressure causes disturbances in the bacterial outer membrane, resulting in a sharp increase in OMV production (Toyofuku et al., 2019), during this process, CA in the environment may experience less obstruction from the biofilm and thus bind to its target more effectively (Kharga et al., 2023). TEM visualization showed that vesicles released under the combined intervention of CA and MEM exhibited deformation and leakage. Changes in OMVs integrity further affect the delivery of virulence factors. Highly intact OMVs can protect their contents from degradation and directly transport virulence factors to target host cells, simultaneously, larger OMVs have a greater binding site surface area (such as LPS), which enables them to absorb antimicrobial compounds and provides more internal volume to encapsulate virulence factor and antibiotic-degrading enzymes (Li et al., 2024). To further elucidate the OMV-based anti-infective pathway of CA, we intranasally infected mice with the collected vesicles (KOMV, KOMV-MEM, KOMV-MEM-CA) and used CA as a therapeutic agent (KOMV-MEM + CA). The results showed that KOMV-MEM caused higher mortality and stronger immune responses, whereas both KOMV-MEM-CA mice and those treated with subcutaneous CA injections after KOMV-MEM infection showed increased survival rates and immune suppression. In particular, CA reduced the iNOS level in lung tissue and elevated the IL-10 level. iNOS is highly expressed mainly in M1-type (classically activated) macrophages, neutrophils, and other immune cells, and is involved in the amplification of inflammatory responses (Paolucci et al., 2023). IL-10 is primarily expressed in M2-type (alternatively activated) macrophages, regulatory T cells, and regulatory B cells, and is closely related to anti-inflammation, tissue repair, and immune suppression (Miki et al., 2021). Their activation levels not only indicate the polarization status of resident or recruited macrophages in the tissue but also reflect the balance of inflammatory response and immune regulation (Tao et al., 2024).

The protein profiles of OMVs are highly consistent with those of the source bacteria, including outer membrane and inner membrane proteins (Hua et al., 2022). SDS-PAGE results of the three types of vesicles revealed a distinctly overexpressed band at approximately 60 kDa in KOMV-MEM, identified as GroEL through proteomic analysis. Traditionally, GroEL has been viewed as a cytoplasmic chaperone. Recent research has indicated that GroEL preferentially localises to the cell membrane under antibiotic-induced heat shock responses, likely serving as a compensatory mechanism for LPS modifications or stress on the outer membrane proteins (Cahill et al., 2015). Such perturbations in the cell membrane facilitate OMV generation (McBroom and Kuehn, 2007). On the other hand, surface GroEL has been shown to promote inflammation and phagocytosis (Zhu et al., 2013), which may be related to GroEL being recognized by the macrophage surface receptor LOX-1, accelerating LPS capture and inducing the initiation of the pyroptosis program (Ye et al., 2021). This research focuses on CA’s inhibition of the pyroptosis and inflammatory cascade triggered by the OMVs-host cell interaction, with macrophages being the representative effector cells in this process (Rankin and Artis, 2018). One of the key mechanisms explored in this study is how the GroEL-LOX-1 interaction drives macrophage uptake of OMVs. We used Alphafold 3 to predict the interaction model between GroEL and LOX-1, and employed molecular docking to predict the potential intervention targets of CA on the interacting proteins. On the other hand, in cell experiments, we confirmed that KOMV-MEM induces more severe macrophage pyroptosis, and the activation of the pyroptosis pathway is highly related to the capture of KOMV-MEM, whereas KOMV-MEM-CA induced pyroptosis was not significant. Free CA has a protective effect on macrophages in a concentration-dependent manner, which is speculated to be related to CA altering membrane structure or inhibiting the pairing of GroEL and LOX-1.

Under antibiotic stress, bacteria express resistance genes and transfer them within or across species to boost adaptability (McEwen and Collignon, 2018). The evolution of bacterial resistance increases the risk of long-term colonisation and secondary infections (Tseng et al., 2017). Bacterial OMVs facilitate the transfer of resistance genes without direct bacterial adhesion, even in the presence of DNase (Johnston et al., 2023). Existing studies have visualized vesicle-mediated DNA delivery events at the single-cell level (Fulsundar et al., 2014). OMVs are akin to nanoscale versions of the originating bacteria and possess a high degree of consistency in content, membrane proteins, and the bacteria themselves (Roier et al., 2016b). The specific mechanism of OMV-mediated plasmid DNA transfer has not been fully elucidated. Existing explanations mainly include conjugation, natural transformation, and transduction, with OMV-mediated transfer being listed as the fourth mechanism (Roier et al., 2016b). Multiple drivers could be involved in the release and recruitment of OMVs, such as the known adhesion and conjunction mediated by OmpA or LPS (Manoil and Rosenbusch, 1982) or the binding of T6SS secreted specificity recognition effector Reut-A1725 (TeoL) with LPS, which is captured by outer membrane receptors, such as CubA or CstR (Li et al., 2022). Prior studies emphasize the importance of TraI and TraN in mediating plasmid DNA transfer. TraI has excellent helicase activity, and the rate of TraI translocation along ssDNA is closely related to the rate of ssDNA transfer to recipient cells (Breidenstein et al., 2023). TraN may pair with various OM proteins in the recipient, which helps enhance the stability of high-risk plasmid transfer (Low et al., 2022). At the molecular level, we predicted the potential targets of CA intervention in the TraI-ssDNA and TraN-OmpK36 complexes. Additionally, Using dual fluorescence signals, we noted a significantly lower conjugation coefficient between KOMV-MEM-CA and sensitive bacteria compared with KOMV-MEM. Under laboratory conditions, KOMV-MEM induced resistance in MEM-sensitive bacteria within 3 h, a process effectively blocked by CA. KOMV-MEM-CA harvested in the presence of both CA and MEM or supplemented with free CA during transmission (KOMV-MEM + CA) inhibited resistance transmission.

During cross-infection with multiple bacterial species, antibiotic pressure not only promotes the transfer of resistance genes between bacterial communities via OMVs, but also enables OMVs carrying various resistance enzymes to hydrolyze antibiotics, thereby protecting susceptible bacteria (Zhang et al., 2023). Although CA lacks significant direct antibacterial activity, this study confirmed that CA can inhibit KOMV-MEM associated resistance enzymes, thus depriving MEM-susceptible bacteria (ATCC700603) of protection and leading to their subsequent hydrolysis by antibiotics. Subsequently, we intranasally infected mice with ATCC700603 + KOMV-MEM and treated them with MEM or MEM combined with CA. The results confirmed the protective effect of KOMV-MEM on susceptible bacteria *in vivo*, as well as the consumption of KOMV-MEM resistance enzymes by CA.

## 5 Conclusion

**FIGURE 8:**
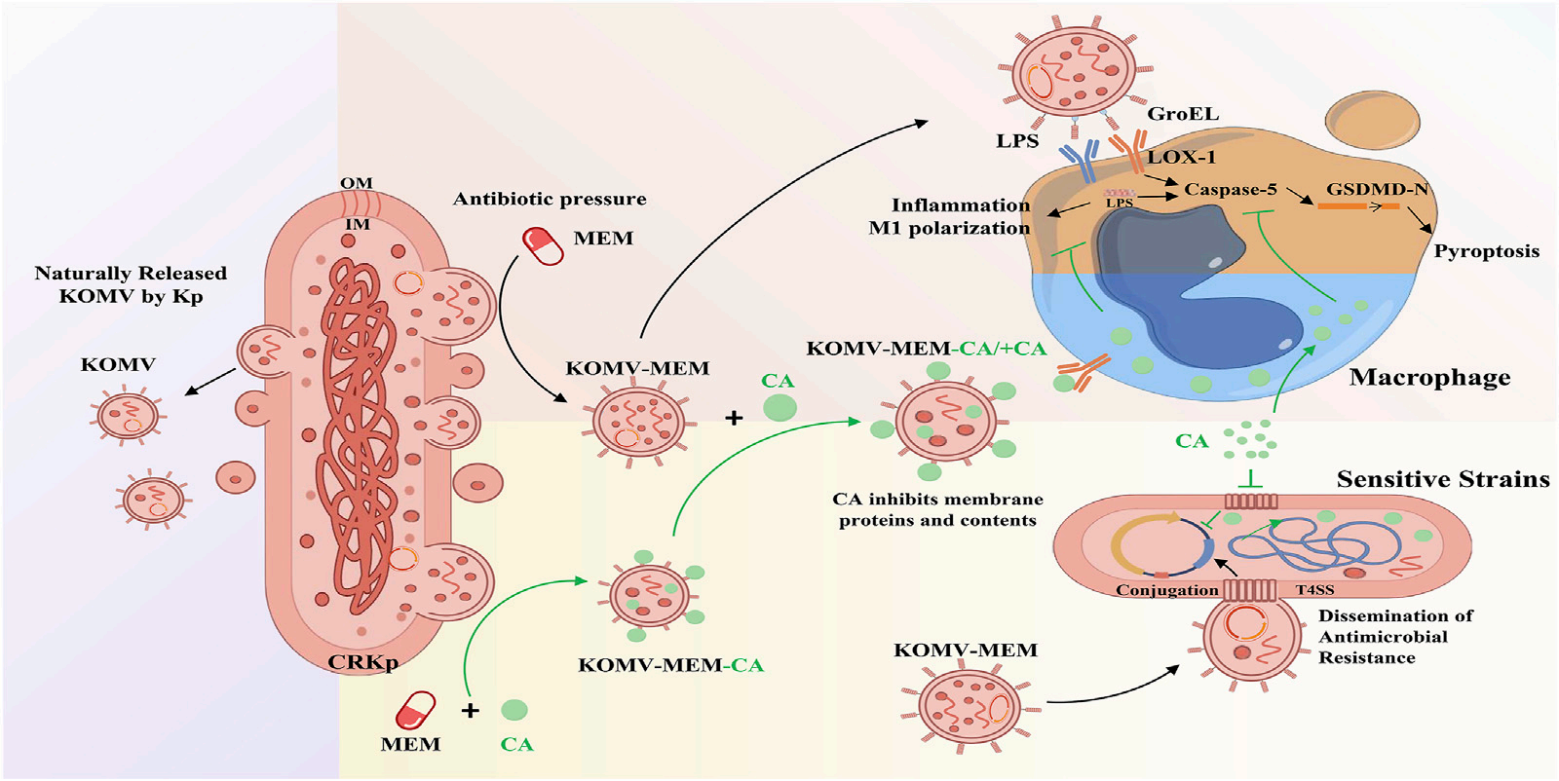
Mechanism diagram of CA inhibiting KOMV-MEM mediated resistance gene transfer and macrophage pyroptosis.

In summary, this study is the first to propose a potential new antibacterial mechanism of CA, which involves inhibiting the virulence and resistance transmission of KOMV-MEM released by CRKp under MEM pressure (Figure 8). The results revealed the potential of CA to reduce the infection risk of CRKP via the OMV pathway.

## Data Availability

The datasets presented in this study can be found in online repositories. The names of the repository/repositories and accession number(s) can be found below: https://www.ncbi.nlm.nih.gov/genbank/, PP348266.

## Abbreviations

CA, chlorogenic acid; CRKp, carbapenem-resistant *klebsiella pneumoniae*; hvKp, highly virulent *Klebsiella pneumoniae*; hv-CRKp, highly virulent CRKp; IN, Intranasal administration; KOMV, OMVs naturally released by Kp694; KOMV-MEM, OMVs released by Kp694 under sub-lethal concentration of MEM pressure; KOMV-MEM + CA, Following the action of KOMV-MEM, CA intervention was further introduced; KOMV-MEM-CA, OMVs released by Kp694 under sub-lethal MEM pressure with CA intervention; MEM, meropenem; MDR, Multidrug Resistance; MØ, Macrophage; OMVs, Outer membrane vesicle; PO, Oral administration; SC, Subcutaneous injection.
