# Supplementary material for: Chlorogenic acid inhibits virulence and resistance gene transfer in outer membrane vesicles of carbapenem-resistant Klebsiella pneumoniae
Source: Front Pharmacol. 2025 Mar 31;16:1562096. doi: 10.3389/fphar.2025.1562096 (PMC11994928; doi:10.3389/fphar.2025.1562096)
Supplement: Supplementary file 1 [file Table1.docx]

**Supplementary Table Primers list**

| Target | Primer | Sequence (5’-3’) |
| --- | --- | --- |
| blaKPC | KPC-F | TCGCTAAACTCGAACAGG |
|  | KPC-R | TTACTGCCCGTTGACGCCCAATCC |
| blaNDM-1 | NDM-F | TTGGCCTTGCTGTCCTTG |
|  | NDM-R | ACACCAGTGACAATATCACCG |
| blaOXA-48(D) | GES-F | CTATTACTGGCAGGGATCG |
|  | GES-R | CCTCTCAATGGTGTGGGT |
| Kp-GroEL | Kp-GroEL-F | CGGTGAAGAGCCGTCTGTTGT |
|  | Kp-GroEL-R | GACTTTGGTTGGGTCCAGGATA |
| l6-S | l6-S-F | TTCGGTCCAGTTGCCTTCTC |
|  | l6-S-R | AGTGCCTCTTTGCTGCTTTCA |
| H-OLR1 | H-OLR1-F | CTGCGACTCTAGGGGTCCTTT |
|  | H-OLR1-R | CTTCCGAGCAAGGGTTTCTATC |
| NLRP3 | NLRP3-F | GCGATCAACAGGAGAGACCTT |
|  | NLRP3-R | TCCACTCCTCTTCAATGCTGT |
| Caspase-5 | Caspase-5-F | CATTACGGAACTCATCACA |
|  | Caspase-5-R | TGCCAGGAAAGAGGTAG |
| GAPDH | GAPDH-F | GGAAGCTTGTCATCAATGGAAATC |
|  | GAPDH-R | TGATGACCCTTTTGGCTCCC |
