# Supplementary figures and images for: Chlorogenic acid inhibits virulence and resistance gene transfer in outer membrane vesicles of carbapenem-resistant Klebsiella pneumoniae
Source: Front Pharmacol. 2025 Mar 31;16:1562096. doi: 10.3389/fphar.2025.1562096 (PMC11994928; doi:10.3389/fphar.2025.1562096)

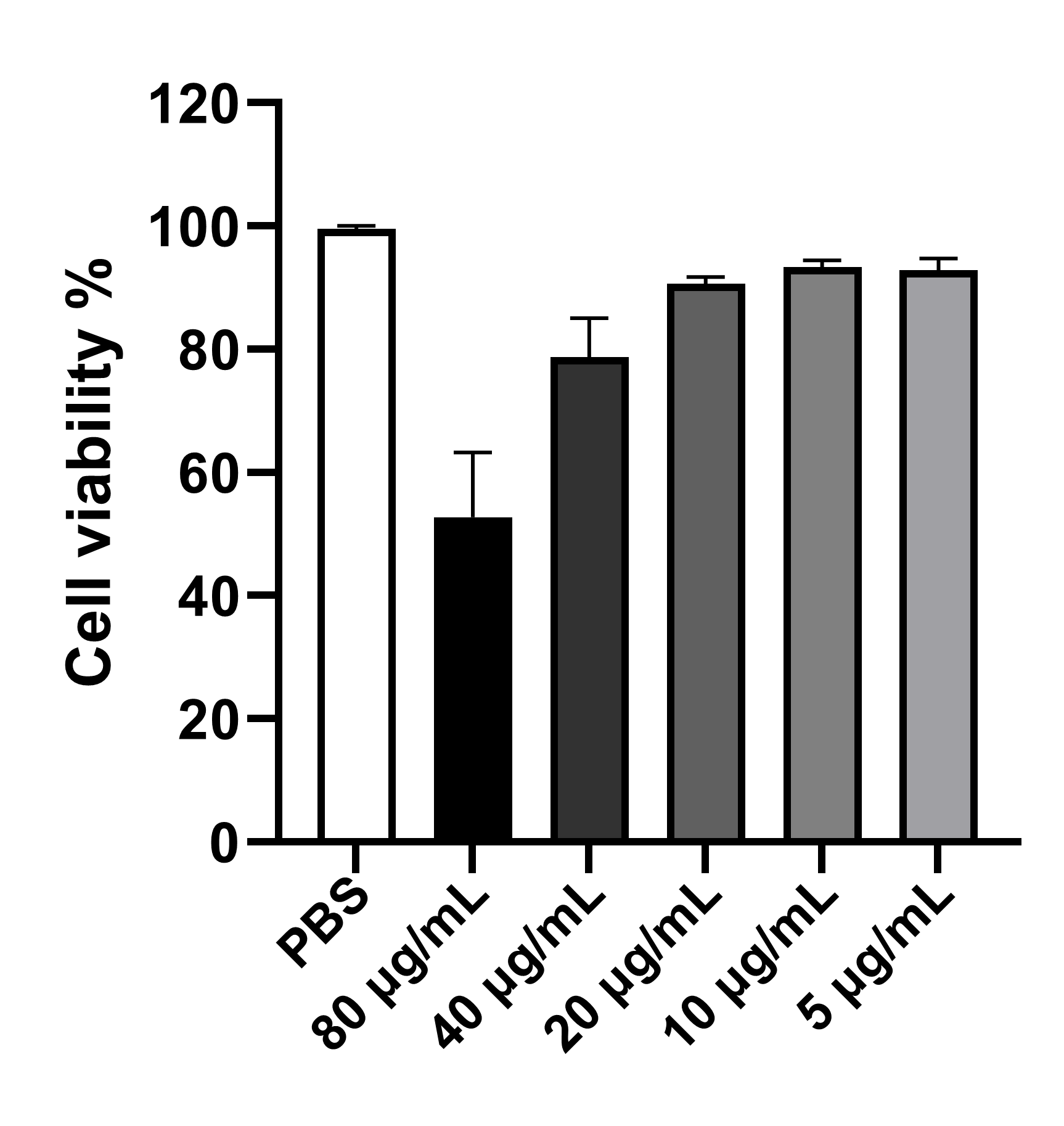

Supplement: Supplementary file 2 [file Image2.tif]

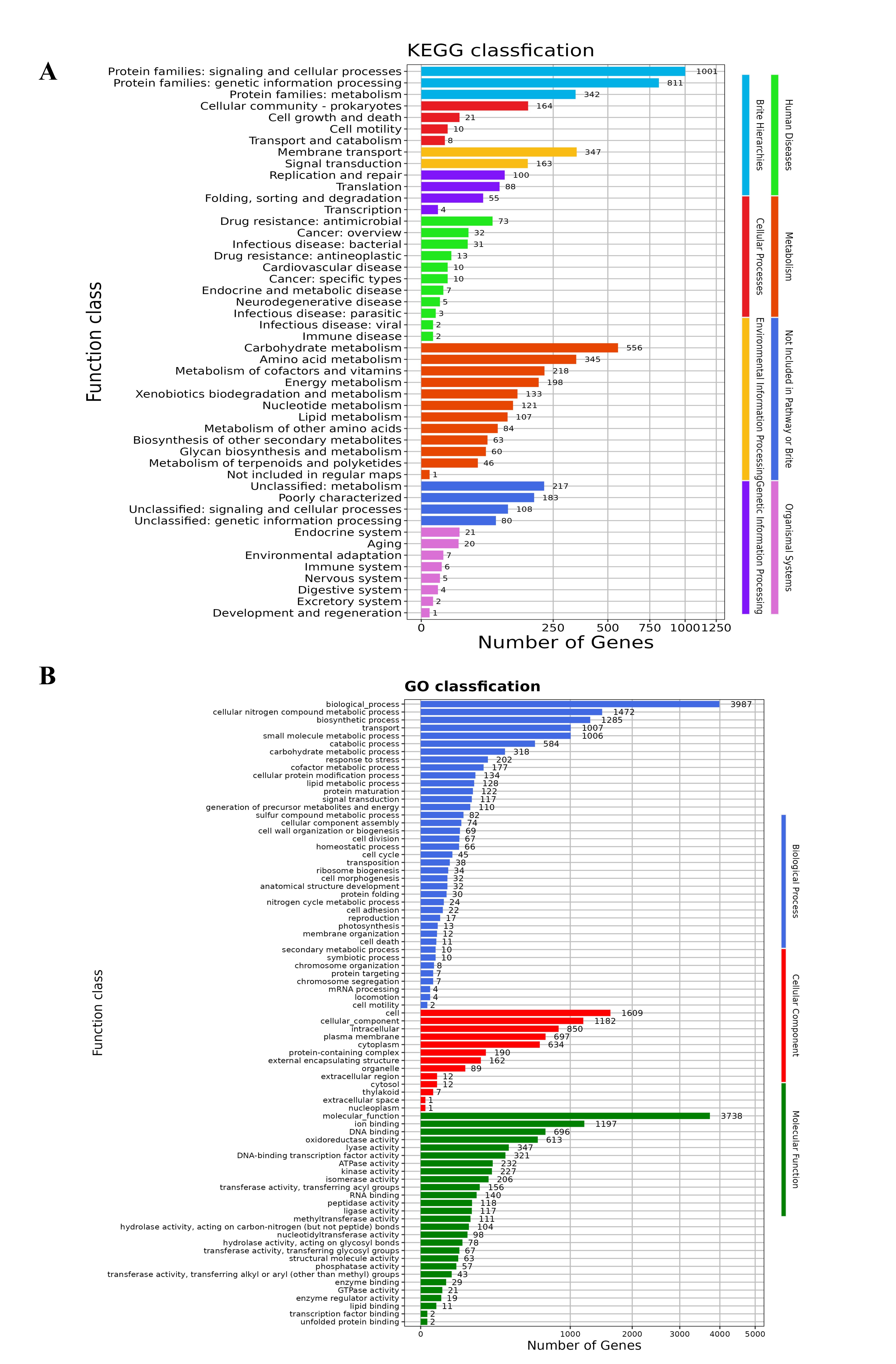

Supplement: Supplementary file 3 [file Image1.tif]
